# Neuropeptide Signaling Differentially Affects Phase Maintenance and Rhythm Generation in SCN and Extra-SCN Circadian Oscillators

**DOI:** 10.1371/journal.pone.0018926

**Published:** 2011-04-29

**Authors:** Alun T. L. Hughes, Clare Guilding, Hugh D. Piggins

**Affiliations:** Faculty of Life Sciences, University of Manchester, Manchester, United Kingdom; Vanderbilt University, United States of America

## Abstract

Circadian rhythms in physiology and behavior are coordinated by the brain's dominant circadian pacemaker located in the suprachiasmatic nuclei (SCN) of the hypothalamus. Vasoactive intestinal polypeptide (VIP) and its receptor, VPAC_2_, play important roles in the functioning of the SCN pacemaker. Mice lacking VPAC_2_ receptors (*Vipr2^−/−^*) express disrupted behavioral and metabolic rhythms and show altered SCN neuronal activity and clock gene expression. Within the brain, the SCN is not the only site containing endogenous circadian oscillators, nor is it the only site of VPAC_2_ receptor expression; both VPAC_2_ receptors and rhythmic clock gene/protein expression have been noted in the arcuate (Arc) and dorsomedial (DMH) nuclei of the mediobasal hypothalamus, and in the pituitary gland. The functional role of VPAC_2_ receptors in rhythm generation and maintenance in these tissues is, however, unknown. We used wild type (WT) and *Vipr2^−/−^* mice expressing a luciferase reporter (PER2::LUC) to investigate whether circadian rhythms in the clock gene protein PER2 in these extra-SCN tissues were compromised by the absence of the VPAC_2_ receptor. *Vipr2^−/−^* SCN cultures expressed significantly lower amplitude PER2::LUC oscillations than WT SCN. Surprisingly, in *Vipr2^−/−^* Arc/ME/PT complex (Arc, median eminence and pars tuberalis), DMH and pituitary, the period, amplitude and rate of damping of rhythms were not significantly different to WT. Intriguingly, while we found WT SCN and Arc/ME/PT tissues to maintain a consistent circadian phase when cultured, the phase of corresponding *Vipr2^−/−^* cultures was reset by cull/culture procedure. These data demonstrate that while the main rhythm parameters of extra-SCN circadian oscillations are maintained in *Vipr2^−/−^* mice, the ability of these oscillators to resist phase shifts is compromised. These deficiencies may contribute towards the aberrant behavior and metabolism associated with *Vipr2^−/−^* animals. Further, our data indicate a link between circadian rhythm strength and the ability of tissues to resist circadian phase resetting.

## Introduction

It is now well-established that the main mammalian circadian pacemaker is localized to the suprachiasmatic nucleus (SCN; [1], [2]). The SCN controls the daily timing of behavioral and physiological processes such as rodent wheel-running and plasma corticosterone [3]. Such intrinsic timekeeping emerges through the synchronized activities of several thousand SCN neurons which themselves function as cell autonomous circadian oscillators [4], [5]. The neuropeptide vasoactive intestinal polypeptide (VIP) is synthesized by neurons in the ventral aspect of the SCN [6], [7], while its cognate receptor, VPAC_2_, is expressed by many neuronal cell types in this structure [8], [9]. Pharmacological studies in wild-type rodents have implicated VIP-VPAC_2_ signaling in the resetting of the SCN pacemaker by light [10], [11] and in setting pacemaker period [12], but the development of transgenic mouse models with impaired VIP-VPAC_2_ expression has revealed a more fundamental role of this signaling pathway.

Mice deficient in VIP (*VIP^−/−^*) or lacking VPAC_2_ receptor expression (*Vipr2^−/−^*) have disrupted circadian rhythms in wheel-running activity, body temperature and sleep, as well as metabolic, cardiovascular, cognitive and endocrine dysfunction [13]–[24]. Such alterations in whole animal behavior and physiology are accompanied by reductions in the synchrony and amplitude of electrical and molecular oscillations of SCN neurons [25]–[29]. Collectively, these studies establish that VIP-VPAC_2_ signaling is necessary for appropriately synchronized high amplitude rhythms in SCN cellular activities and circadian control of brain, body, and behavior [30].

The mammalian circadian system was once conceptualized as consisting of the main SCN pacemaker, whose outputs organized rhythmic activity in downstream effector sites. Accordingly, this uniclock view did not afford extra-SCN brain sites or peripheral tissues with significant endogenous circadian oscillatory capabilities. However, with the determination of the molecular basis of SCN timekeeping (the so-called core clock genes such as *per1-2*, *cry1-2*, *bmal1*, etc; [31]–[33]) and the demonstration that these genes/proteins are rhythmically expressed in extra-SCN tissues, including the liver, adrenal and pituitary glands [34]–[41], this uniclock model is now known to be incorrect [42], [43]. The development of fluorescent and bioluminescent reporters of clock genes and proteins now allows assessment of the capacity of a tissue to generate circadian rhythms when isolated in culture, independent of SCN-derived signals [44]–[46]. Using the *mPer2^luc^* knockin mouse, in which the expression of PER2 is reported by luciferase, we recently showed that rhythms of PER2 bioluminescence are readily measured in the dorsomedial (DMH) and arcuate (Arc) nuclei, median eminence (ME) and pars tuberalis (PT) of the mediobasal hypothalamus (MBH; [47]); areas intimately involved in the control of metabolism [48], [49]. This complemented earlier studies in this mouse model reporting robust PER2::LUC expression in peripheral tissues including the pituitary gland [46]. Since VIP is synthesized in the pituitary [50], [51], VIP-ir terminals are present in the MBH [52], [53] and VIP binding sites/VPAC_2_ mRNA are present in the DMH, Arc, and pituitary [8], [54], [55], we investigated whether circadian rhythms in PER2::LUC bioluminescence in these extra-SCN tissues were compromised by the absence of the VPAC_2_ receptor.

## Methods

### Ethics Statement

All experiments were performed in accordance with the UK Animals (Scientific Procedures) Act of 1986 using procedures approved by The University of Manchester Review Ethics Panel.

### Animals and Behavioral Analysis

For this study, *mPer2^luc^* mice [46] were cross-bred with *Vipr2^−/−^* mice [13] to generate a PER2::LUC reporter strain that lacked expression of functional VPAC_2_ receptors (*mPer2^luc^*×*Vipr2^−/−^*; herein referred to as *Vipr2^−/−^*). Standard *mPer2^luc^* mice (expressing functional VPAC_2_ receptors) from the University of Manchester breeding colony were used as controls (WT). All mice used in this study were adult males on a C57BL/6 background, housed at 20–22°C and humidity ∼40%, with *ad libitum* access to food and water.

Animals were initially bred and maintained group housed under a 12 h light∶12 h complete darkness cycle (LD; Zeitgeber time [ZT] 0 was defined as the time of lights on). Animals contributing to the LD part of the study were taken directly from these conditions and culled during the mid-late day phase (mean cull phase ZT6.7±1.0 h). Behaviorally screened mice were singly housed in running wheel-equipped cages (wheel diameter 16 cm) under LD for at least 7 days then transferred to constant darkness (DD) for at least 14 days before cull. Analyses of period and rhythm strength (percentage of variance accounted for by the rhythm; %V) of wheel-running activity for mice in DD were made using actograms and *Chi^2^* periodograms created with the Analyze9 software package (Stanford Software Systems, Santa Cruz, CA) on the final 14 days before cull. Using the onset of wheel-running activity as a phase marker (circadian time [CT] 12 was defined as the onset of the daily activity bout), DD mice were culled at times spanning the circadian cycle to allow assessment of the effect of culture preparation time on the phase of peak PER2::LUC expression. *Vipr2^−/−^* mice that did not express a significant circadian rhythm were culled at arbitrary timepoints. Mice were classified as either rhythmic or expressing multiple low power rhythmic components (arrhythmic) according to previously defined criteria [56].

### Culture Preparation and Luminometry

Mice were culled by cervical dislocation following isoflurane anaesthesia (Baxter Healthcare Ltd, Norfolk, UK). SCN, Arc/ME/PT complex (combined) and DMH were micro-dissected and cultured as 300 µm thick coronal slices (cut from a consistent rostro-caudal level for each tissue, based on neuroanatomical landmarks and the Paxinos and Franklin mouse brain atlas [57] as previously described [47], [58]. Pituitaries were removed by hand and cultured whole, under identical conditions. Night-vision goggles were used during DD culls to maintain darkness until mice had been euthanased and enucleated.

Brain and pituitary cultures were maintained at 37°C in light-tight incubators (Galaxy R+, RS Biotech, Irvine, Scotland) and total PER2::LUC bioluminescence emission recorded for at least 7 days using photomultiplier tube (PMT) assemblies (H8259/R7518P; Hamamatsu, Welwyn Garden City, UK). Emitted photon counts were integrated for 59 s every 1 min and raw bioluminescence data were processed by subtracting a 24 h running mean to remove long term trends (baseline-subtracted) then smoothed with a 3 h running average. The longitudinal study design employed here allows sensitive identification of low amplitude rhythms in individual animals, such as those of *Vipr2^−/−^* mice. Discontinuous sampling methods, which assess population level trends across a number of individuals, can fail to detect significant variation when individuals are not synchronized to one another or peak-trough amplitude is low [13], [17], [59].

### Gastrin Releasing Peptide and Forskolin Treatments

To investigate the effects of gastrin releasing peptide (GRP) and adenylate cyclase signaling on circadian expression of PER2::LUC in WT and *Vipr2^−/−^* tissues, cultures were treated with either 100 nM GRP (Tocris Bioscience, Bristol, UK) or 10 µM forskolin (an adenylate cyclase activator; Sigma, Poole, UK) 3–8 days following culture preparation. Drugs were administered as complete medium changes to fresh culture medium containing the drug but otherwise identical to control medium.

### Data Analysis and Statistics

Rhythmic bioluminescence traces were assessed by two experienced, independent researchers, blinded to conditions, to extract the following parameters: period, amplitude and phase of peak bioluminescence expression. Period was assessed using peak-peak and trough-trough durations averaged over as many cycles as possible for each individual tissue explant, discounting the first 24 h of data. At least one full peak-peak or trough-trough cycle was assessed of each explant, though in the majority of cases two or more full cycles were used. Amplitude was measured as the peak-trough difference 24–48 h after culture preparation from baseline-subtracted traces and phase was assessed as the time of the first peak to occur between 24–48 h after culture preparation. Further, the rate of damping of PER2::LUC bioluminescence rhythms was assessed as the number of cycles before smoothed bioluminescence variation reached the amplitude of dark noise (previously determined for each PMT module). The projected rate of damping was calculated for cultures that showed obvious damping of their bioluminescence rhythm but had not fully damped by the end of data acquisition. Statistically significant differences between genotypes were determined using unpaired *t*-tests performed in Microsoft Excel (*p*<0.05 required for significance). Rayleigh vector plots (custom software designed in house by Dr T. Brown and El Temps, Dr. A. Díez-Noguera, Barcelona, Spain) were used to assess the significance of phase clustering of peak phases in relation to circadian time (CT) or to time from culture preparation. Responses to forskolin and GRP treatments were assessed by comparison of amplitude 24 h before and 24 h after treatments, not including treatment artifacts. Statistical significance was determined by comparison of amplitude changes between control and drug treatments (2-way analysis of variance with *a priori* pairwise comparisons).

## Results

### Locomotor Activity Rhythms of *Vipr2^−/−^* mice are not Altered by the PER2::LUC Reporter

**Figure 1:**
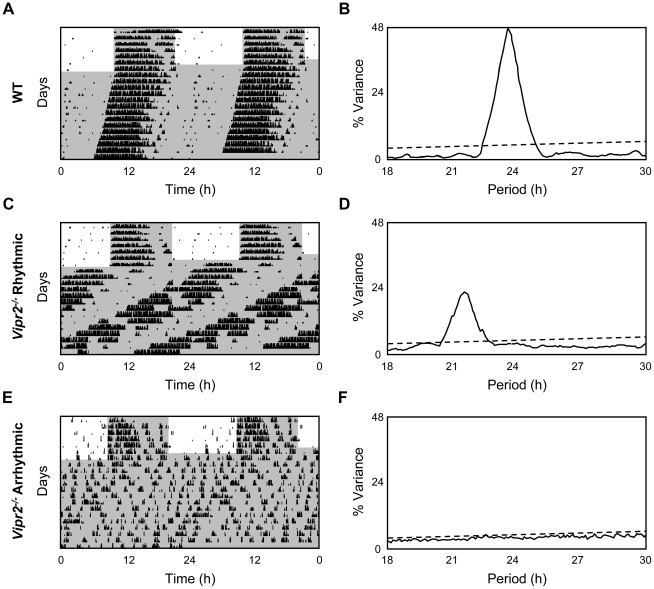
Representative Actograms and Periodograms for Individual WT and *Vipr2^−/−^* Mice Expressing the PER2::LUC Reporter. Both WT and *Vipr2^−/−^* PER2::LUC-expressing mice synchronize to an LD cycle (**A**, **C**, **E**). WT mice [expressing the PER2::LUC reporter] exhibit a strong, near 24 h, locomotor activity rhythm in DD, evident on the actogram (**A**) and from the corresponding high power periodogram peak at ∼24 h (**B**). Activity recordings and periodograms from *Vipr2^−/−^* mice expressing the PER2::LUC reporter display a continuum of behavioral phenotypes in DD, from strongly rhythmic with a shortened behavioral period (∼22.5 h; **C**–**D**) to arrhythmic (**E**–**F**). Actograms are double-plotted, showing 2 days per row; shaded areas on actograms represent darkness. Peridograms depict period (hours; *x* axis) and strength of the rhythm (%V; *y* axis). Dashed line indicates *p* = 0.001.

*mPER2^luc^* (WT) mice behaved in a manner consistent with previous reports, both for this strain [46] and non-*mPER2^luc^* WT mice (e.g. [20], [60], [61]). All WT mice (n = 9) entrained to the LD cycle, confining activity to the dark phase, and all free ran with a strong circadian rhythm in DD (mean period 23.82±0.05 h and rhythm strength (%V) of 58.51±5.34%), starting from the time of activity onset under LD (Fig. 1A–1B).

Circadian locomotor activity of *Vipr2^−/−^* mice was not altered by the PER2::LUC reporter transgene; mice expressed locomotor activity rhythms consistent with previous descriptions for non-*mPER2^luc^ Vipr2^−/−^* mice [20], [62]. Briefly, *Vipr2^−/−^* mice (n = 13) limited their activity to the dark period of the LD cycle and on release into DD began activity almost immediately, defining a large phase advance from the timing of LD activity (Fig. 1C, 1E). In DD, *Vipr2^−/−^* mice expressed the continuum of behavioral phenotypes common for this genotype, from robustly rhythmic with a single dominant component of locomotor behavior (Fig. 1C–1D), to apparent arrhythmicity, often with multiple, low power periodic components (Fig. 1E–1F). Approximately 50% (6/13) of *Vipr2^−/−^* individuals were classified as rhythmic in DD expressing a mean period of 22.47±0.63 h and rhythm strength of 24.14±3.70%. Both period and rhythm strength of *Vipr2^−/−^* mice were significantly different to those of WTs (*p*<0.05 and *p*<0.0001, respectively).

### Circadian Rhythms of PER2::LUC Expression are Maintained, but Diminished in the *Vipr2^−/−^* SCN

**Figure 2:**
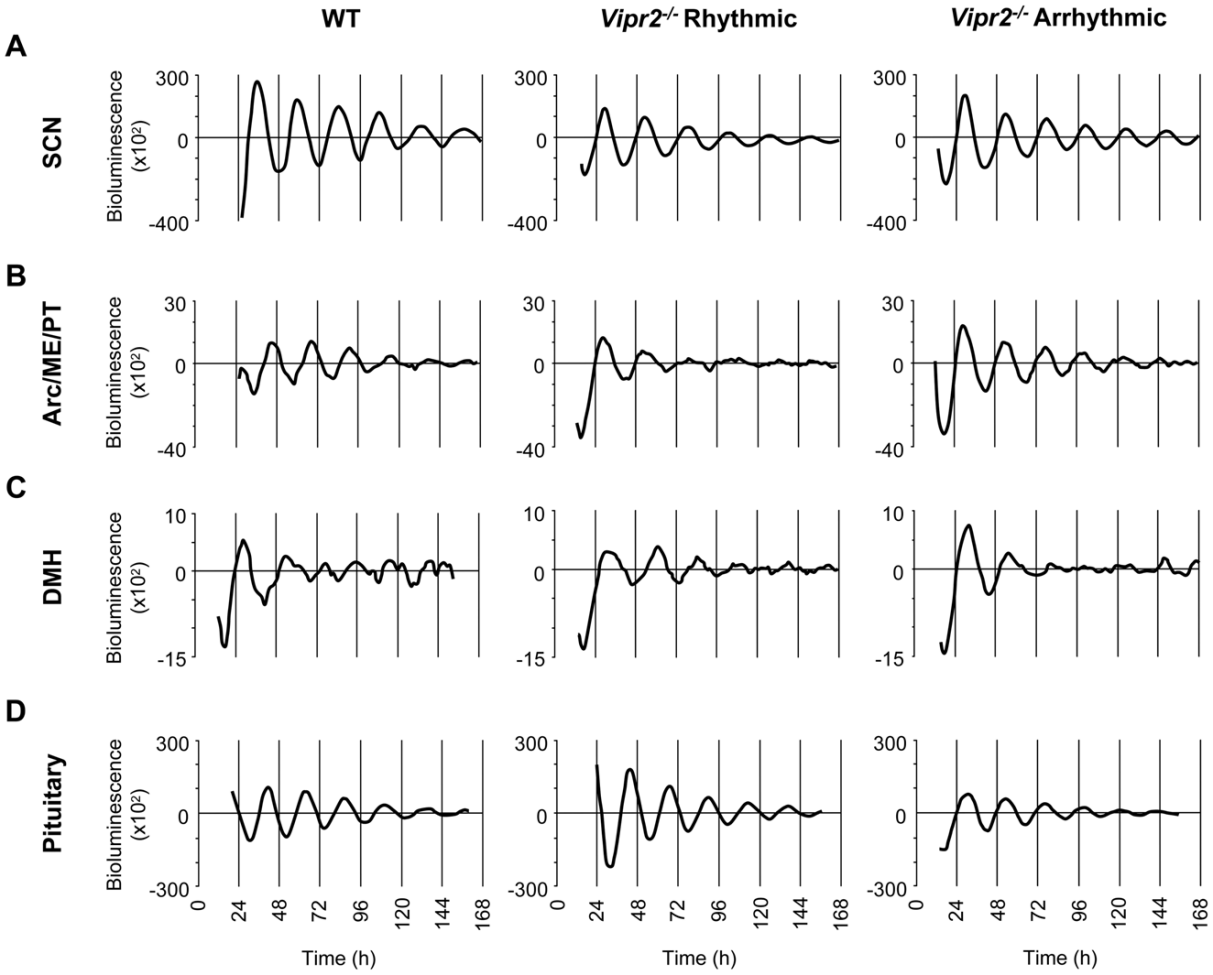
Circadian Rhythms in PER2::LUC Expression in WT and *Vipr2^−/−^* SCN, MBH and Pituitary. Representative plots of detrended PER2::LUC bioluminescence expression from SCN (**A**), Arc/ME/PT complex (**B**), DMH (**C**) and pituitary (**D**) cultures, prepared from behaviorally rhythmic WT animals and from both behaviorally rhythmic and arrhythmic *Vipr2^−/−^* animals all taken from DD free-running conditions. (**A**) The amplitudes of both rhythmic and arrhythmic *Vipr2^−/−^* SCN PER2::LUC rhythms are significantly lower in than WT SCN rhythms. No differences were found in rhythm characteristics between rhythmic and arrhythmic *Vipr2^−/−^* SCN. (**B–D**) No differences were observed between the PER2::LUC rhythms of WT and *Vipr2^−/−^* mice in any circadian parameter assessed. Traces for WT SCN, Arc/ME/PT and pituitary are plotted as circadian time while all other tissues are plotted as time from culture preparation.

All SCN cultures recorded during this study expressed strong circadian rhythms of PER2::LUC bioluminescence that remained robustly rhythmic for the duration of recording (at least 7 days *in vitro*; Fig. 2A). The period of rhythms expressed by *Vipr2^−/−^* SCN cultures did not differ from WT SCN rhythms, in cultures prepared from mice under either LD or DD conditions (both *p*>0.05; Table 1). The amplitude of *Vipr2^−/−^* SCN rhythms was, however, significantly lower than that of WT SCN in cultures from mice housed in both lighting conditions (*p*<0.01 and *p*<0.05 for LD and DD respectively; Table 1; Fig. 2A). No significant difference was found in either period or amplitude between behaviorally screened rhythmic and arrhythmic *Vipr2^−/−^* mice in DD (*p*>0.05; Fig. 2A).

**Table 1:** Bioluminescence Data for Circadian Parameters of WT and *Vipr2^−/−^* Tissues.

		LD		DD	
		WT	*Vipr2^−/−^*	WT	*Vipr2^−/−^*
**SCN**					
	Number of cultures	n = 10	n = 13	n = 9	n = 13
	% Rhythmic	100%	100%	100%	100%
	Period (h)	24.24±0.16	23.81±0.14	24.54±0.22	24.41±0.19
	Rate of Damping (d)	N/A	N/A	N/A	N/A
	Amplitude (arbitrary units)	3129±446	1465±243**	6583±1628	3255±529*
**Arc/ME/PT**					
	Number of cultures	n = 8	n = 13	n = 9	n = 13
	% Rhythmic	88%	85%	89%	100%
	Period (h)	23.34±0.54	23.45±0.28	23.14±0.21	24.11±0.56
	Rate of Damping (d)	4.6±0.6	4.0±0.6	4.0±0.7	3.3±0.5
	Amplitude (arbitrary units)	169±31	145±22	126±30	151±24
**DMH**					
	Number of cultures	n = 7	n = 12	n = 9	n = 13
	% Rhythmic	71%	83%	100%	100%
	Period (h)	25.02±0.92	24.07±0.65	25.22±0.97	24.86±0.62
	Rate of Damping (d)	2.2±0.4	2.1±0.3	2.7±0.7	2.1±0.2
	Amplitude (arbitrary units)	100±35	110±20	130±24	111±15
**Pituitary**					
	Number of cultures	n = 8	n = 13	n = 9	n = 8
	% Rhythmic	100%	100%	100%	100%
	Period (h)	23.28±0.34	23.60±0.12	23.53±0.27	23.63±0.22
	Rate of Damping (d)	N/A	N/A	N/A	N/A
	Amplitude (arbitrary units)	5180±1197	4727±736	3625±690	2486±879

NOTE: LD = tissue collected from mice housed under a 12 h∶12 h light∶dark cycle; DD = tissue collected from mice housed in constant darkness; WT = *mPer2^luc^*-expressing wild type mice; *Vipr2^−/−^* = *mPer2^luc^*-expressing mice lacking functional expression of the VPAC_2_ receptor gene.

* = significant difference to WT under DD at *p*<0.05;

** = significant difference to WT under LD at *p*<0.01.

Values are presented as mean ± SEM.

### Circadian Rhythms of PER2::LUC Expression are Not Compromised in the Mediobasal Hypothalamus and Pituitary of *Vipr2^−/−^* Mice

Consistent with earlier work [44], [46], [47], Arc/ME/PT, DMH, and pituitary cultures from WT mice showed significant circadian oscillations in PER2::LUC bioluminescence (Fig. 2B–2D). Examination of period, amplitude and rate of damping revealed no significant differences between the rhythms of WT and *Vipr2^−/−^* mice expressed in the Arc/ME/PT, DMH or pituitary gland, in tissue collected from mice housed in either LD or DD (Table 1; all comparisons *p*>0.05; Fig. 2B–2D). Further, no significant differences were found in any of these tissues between the rhythms expressed by rhythmic and arrhythmic *Vipr2^−/−^* mice (all *p*>0.05; Fig. 2B–2D).

### The Phases of WT and *Vipr2^−/−^* PER2::LUC Rhythms in MBH Tissues, Pituitary and SCN are Differentially Sensitive to Resetting by Culture Procedure

The phase of peak expression of PER2::LUC was only assessed for cultures collected from mice housed in DD as these individuals were culled at a wide range of times throughout the circadian cycle. This allowed assessment of whether the peak phase of different tissues from WT and *Vipr2^−/−^* mice was associated with a particular CT phase or was reset by cull/culture procedure and expression peaked the same number of hours after cull regardless of the CT phase of cull.

**Figure 3:**
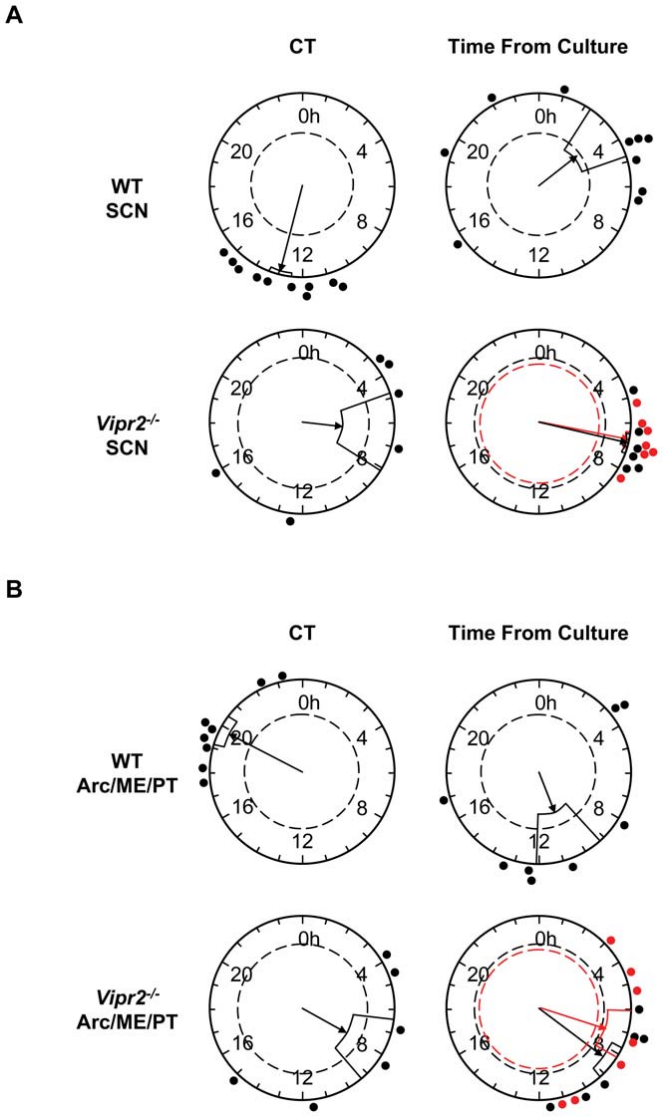
Rayleigh Plots Showing the Effect of Culture Preparation on Peak Phase of PER2::LUC Expression in SCN and Arc/ME/PT. Plots show peak PER2::LUC phase for WT and *Vipr2^−/−^* SCN and Arc/ME/PT plotted as either circadian time (CT; based on behavioral rhythms) or time of peak bioluminescence after culture preparation (Time From Culture). CT plots include data from behaviorally rhythmic mice only (black data points, arrow and dashed line) while time from culture plots show data both from behaviorally rhythmic (black) and arrhythmic (red) mice, analyzed separately. Black data points from behaviorally rhythmic mice on CT plots and time from cull plots are directly comparable. Red points from arrhythmic *Vipr2^−/−^* mice are included for subjective comparison. Both SCN and Arc/ME/PT from WT mice express peak PER2::LUC bioluminescence at a consistent circadian phase, regardless of cull/culture time (peak phase is correlated with CT not with time from culture). However, *Vipr2^−/−^* SCN and Arc/ME/PT always peak the same number of hours after culture preparation, showing these tissues to be reset by this process (peak phase correlated with time from culture not CT. Note that phase is well clustered for WT CT plots (upper left of panels **A** and **B**) and *Vipr2^−/−^* time from culture plots (lower right of panels **A** and **B**). Filled circles indicate the phase of peak bioluminescence in individual cultures. The direction of an arrow indicates the mean phase vector and its length shows significance relative to the (*p* = 0.05) significance threshold indicated by the inner broken circle. Boxes surrounding arrow heads show variance of phase between cultures.

Overall, WT SCN peak phase was significantly associated with CT (*p*<0.00001; Fig. 3A); rhythms peaked at a mean phase of CT12.9±0.5, with a slightly earlier phase (∼CT11–12) observed in cultures from mice culled during the middle of the circadian day, and a slightly later phase (∼CT14) observed in cultures prepared at other times. *Vipr2^−/−^* SCN, however, were reset by cull/culture procedure and PER2::LUC rhythms consistently peaked at ∼31 h after cull (30.7±0.3 h; SCN peak phases from behaviorally rhythmic (30.8±0.5 h) and arrhythmic (30.6±0.4 h) *Vipr2^−/−^* mice combined), regardless of the circadian phase of cull and culture preparation. Indeed, Rayleigh analysis revealed *Vipr2^−/−^* SCN peak phase from rhythmic individuals to correlate significantly with time of cull (*p*<0.0001) and not CT (Fig. 3A). Behaviorally arrhythmic *Vipr2^−/−^* mice were not included in these Rayleigh analyses as CT phase could not be calculated for these individuals, however, a separate analysis of the peak phase for arrhythmic *Vipr2^−/−^* SCN cultures also revealed a significant association with time from cull (Fig. 3A; *p*<0.0001).

Similarly to SCN cultures, WT Arc/ME/PT expressed peak levels of PER2::LUC at a consistent circadian phase (CT19.9±0.7; Rayleigh correlation with CT: *p*<0.0001) and were not reset by cull/culture procedure (Fig. 3B). *Vipr2^−/−^* Arc/ME/PT cultures, as for *Vipr2^−/−^* SCN, were reset by cull/culture and peaked 31.8±0.7 h following cull (behaviorally rhythmic (32.5±0.9 h) and arrhythmic (31.1 h±1.4 h) individuals combined). *Vipr2^−/−^* Arc/ME/PT peak phase for both rhythmic (*p*<0.005) and arrhythmic (*p*<0.05) mice correlated significantly with time from cull (Fig. 3B). While pituitary cultures from WT mice consistently expressed peak levels of PER2::LUC expression at CT17.7±1.4 h (Rayleigh correlation with CT: *p*<0.01), the phase of *Vipr2^−/−^* pituitaries differed widely between individuals and was not consistently associated with either a particular circadian phase or duration of time following cull (Rayleigh associations with CT (for behaviorally rhythmic individuals only) and cull time (rhythmic and arrhythmic individuals separately), all *p*>0.05). DMH tissue from both WT and *Vipr2^−/−^* mice reset to ∼30 h after cull (29.7±1.2 h and 30.1±0.5 h, respectively), regardless of the CT cull phase or behavioral rhythmicity of the animal (Rayleigh correlation with time from cull *p*<0.05, *p*<0.005 and *p*<0.005, respectively for WT, behaviorally rhythmic and behaviorally arrhythmic *Vipr2^−/−^* mice).

### Circadian Rhythms of PER2::LUC Expression in MBH Tissues, Pituitary and SCN are Differentially Affected by Treatments with Forskolin and Gastrin Releasing Peptide

To investigate the health of tissues after rhythms had damped, cultures were treated with the adenylate cyclase activator, forskolin (10 µM), previously shown to boost rhythms in cultured circadian oscillator tissues [44]. Forskolin treatment induced robust increases in rhythm amplitude in tissues that previously showed either a circadian deficit due to altered VIP-VPAC_2_ signaling (*Vipr2^−/−^* SCN; *p*<0.005; Table 2), or demonstrated rapid damping of oscillations (WT and *Vipr2^−/−^* Arc/ME/PT, *p*<0.005 and *p*<0.0005, respectively; WT and *Vipr2^−/−^* DMH, *p*<0.01 and *p*<0.005, respectively; Table 2). More robustly rhythmic tissues (WT SCN, WT and *Vipr2^−/−^* pituitary gland) did not show significant increases in rhythm amplitude following forskolin treatment (Table 2), presumably as these tissues had not yet significantly damped at the time of treatment and/or did not suffer any inherent rhythm abnormalities associated with a lack of VPAC_2_ receptors.

**Table 2:** Bioluminescence Amplitude Data for Responses of WT and *Vipr2^−/−^* Tissues to Control and GRP Treatments.

	WT			*Vipr2^−/−^*		
	Control	Forskolin	GRP	Control	Forskolin	GRP
**SCN**	−1206±399 (4)	−2080±417 (3)	−1477±430 (6)	−225±225 (4)	1571±409*** (4)	−408±339 (4)
**Arc**	−20.6±12.3 (4)	143±36*** (4)	61.8±26.2* (4)	−34.2±22.8 (6)	448±92**** (4)	59.5±29.6 * (4)
**DMH**	−10.0±10.7 (4)	86±29** (5)	91.7±48.1* (5)	−0.7±3.2 (4)	133±29*** (4)	7.8±9.4 (4)
**Pituitary**	105±215 (4)	−701±658 (5)	695±226† (5)	−477±461 (4)	−1367±582 (4)	−243±631 (4)

NOTE: Values are presented as mean amplitude change from pre- to post-treatment ± SEM. All treatments were delivered to tissue collected from mice housed under a 12 h∶12 h light∶dark cycle. WT = *mPer2^luc^*-expressing wild type mice; *Vipr2^−/−^* = *mPer2^luc^*-expressing mice lacking functional expression of the VPAC_2_ receptor gene.

* = significant difference to control treatment at *p*<0.05;

** = significant difference to control treatment at *p*<0.01;

*** = significant difference to control treatment at *p*<0.005;

**** = significant difference to control treatment at *p*<0.0005;

† = GRP-treated WT pituitary responses were not significantly different to control responses (as some control-treated WT pituitaries showed an increase in rhythm amplitude), however, GRP did induce a significant increase (*p*<0.05) in post-treatment vs. pre-treatment amplitude.

**Figure 4:**
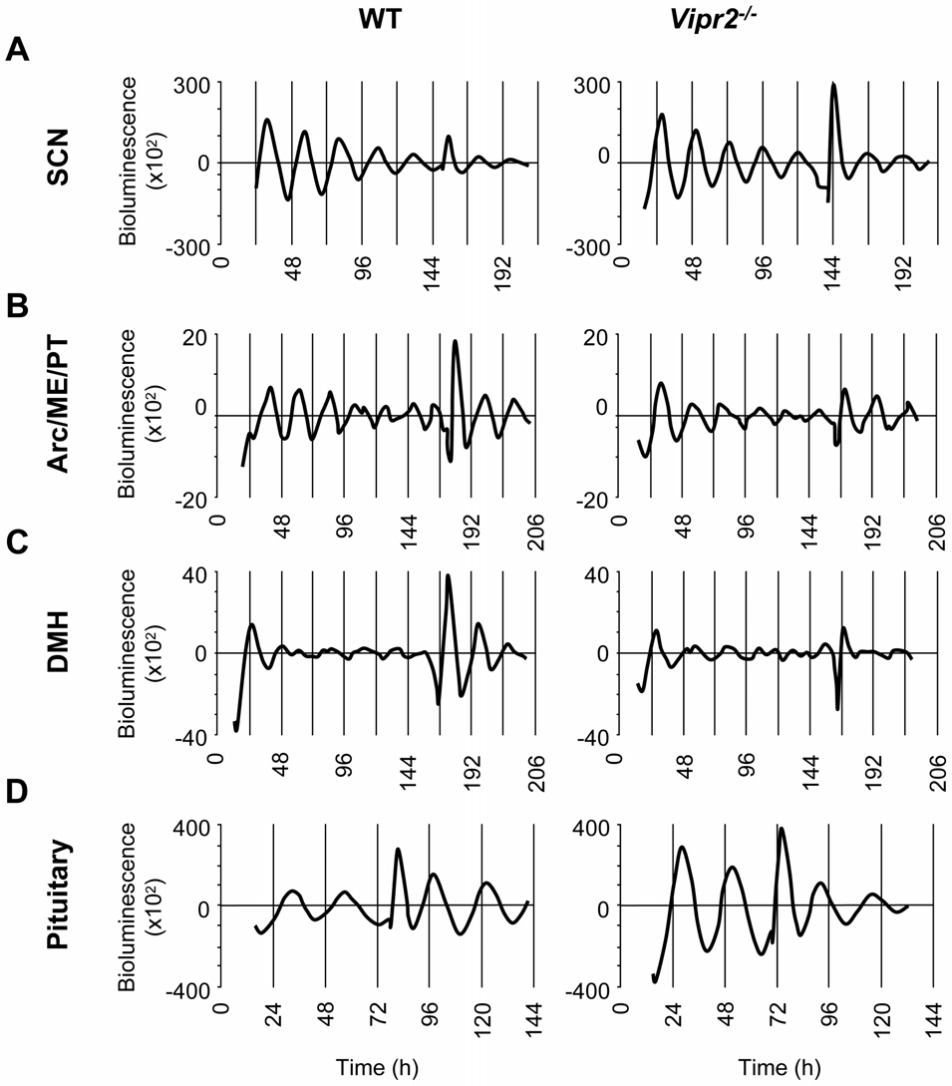
Rhythm Amplitude of PER2::LUC Expression in WT and *Vipr2^−/−^* SCN, MBH and Pituitary are Differentially Affected by GRP Treatment. Representative plots of detrended PER2::LUC bioluminescence expression from WT and *Vipr2^−/−^* SCN (**A**), Arc/ME/PT complex (**B**), DMH (**C**) and pituitary (**D**) cultures, prepared from mice housed under LD conditions. (**A**) GRP treatment failed to increase rhythm amplitude in both WT and *Vipr2^−/−^* SCN. (**B**) Both WT and *Vipr2^−/−^* Arc/ME/PT responded to GRP treatment with an increase in rhythm amplitude. (**C**) GRP application increased the amplitude of DMH rhythms in WT tissue but not *Vipr2^−/−^*. (**D**) WT pituitaries responded to GRP application with an increase in rhythm amplitude (though see results text and Table 2 note) while *Vipr2^−/−^* pituitaries failed to do so. Traces for WT SCN, Arc/ME/PT and pituitary are plotted as circadian time while all other tissues are plotted as time from culture preparation. Note treatment artefacts immediately after GRP application to cultures.

Negative values arise due to the normal reduction in rhythm amplitude over time (e.g. see Figs. 2 and 4) when treatments do not induce an increase in amplitude. Numbers in brackets indicate ‘n’ contributing to that data value.

GRP treatment to *Vipr2^−/−^* SCN tissue *in vitro* has been shown to boost and resynchronize rhythms [27], [29] and GRP receptors are expressed in the pituitary gland and arcuate nucleus [63], [64]. We, therefore, investigated whether GRP treatments would induce similar responses from oscillators in the MBH and pituitary. Surprisingly, neither WT nor *Vipr2^−/−^* SCN rhythms were markedly improved by treatment with 100 nM GRP (both *p*>0.05; Table 2, Fig. 4A). The amplitude of Arc/ME/PT rhythms, however, was increased by treatment with GRP (*p*<0.05 for both WT and *Vipr2^−/−^* tissue; Table 2, Fig. 4B). Interestingly, though GRP receptor expression has not been reported in the DMH, WT DMH cultures showed an increase in rhythm amplitude in response to GRP treatment (*p*<0.05; Table 2, Fig. 4C) while *Vipr2^−/−^* DMH cultures failed to do so. The amplitude change of rhythms expressed by WT pituitaries in response to GRP was not significantly different to control treatments (Table 2, Fig. 4D), however, this lack of significance resulted from control treatments inducing an increase in amplitude in 3 of 5 cultures. Indeed, GRP treatments did induce a significant increase in amplitude in WT pituitary cultures (post-treatment amplitude vs. pre-treatment amplitude *p*<0.05; Table 2, Fig. 4D) and the mean increase in amplitude in response to GRP treatment was 665% of the mean control treatment-induced response. Conversely, neither GRP nor control treatment increased the amplitude of PER2::LUC expression in *Vipr2^−/−^* pituitaries (*p*>0.05; Table 2, Fig. 4D).

## Discussion

### Clock Gene Rhythms Persist in the *Vipr2^−/−^* MBH and Pituitary

While the SCN innervates the MBH region [53], endogenous rhythms in clock gene expression are present *in vitro* in the MBH and pituitary gland [44], [46], [47], [65], tissues critical for the regulation of metabolism [49]. Signaling via the VPAC_2_ receptor is essential for the generation of high amplitude synchronized SCN clock gene rhythms [13], [26], [27] and for appropriate temporal control of behavior and metabolism [18], [20]. Here, we demonstrate that, despite known alterations of circadian function in *Vipr2^−/−^* mice, the lack of VPAC_2_ receptors does not alter period, amplitude or rate of damping of rhythms in PER2 expression in the *Vipr2^−/−^* MBH and pituitary. These rhythms were observed in slice cultures from mice housed in LD and persisted in tissues collected from mice housed in DD, despite the behavioral arrhythmicity of ∼50% of *Vipr2^−/−^* mice in constant conditions. This study provides the first description of extra-SCN neural oscillators in mice lacking either VIP or VPAC_2_, while our demonstration of rhythm maintenance in the *Vipr2^−/−^* pituitary gland concurs with a previous report of peripheral oscillations in the liver and heart of these mice [66].

### *Vipr2* ^−/−^ SCN Phase is Reset by Culture Procedure: Oscillator Strength Determines the Ability of Tissues to Maintain In Vivo Phase when Cultured *In Vitro*

Consistent with previous investigations, both in our laboratory and elsewhere, *Vipr2^−/−^* SCN were found to express significantly lower amplitude clock gene oscillations than WT SCN [25]–[27]. In contrast, the MBH and pituitary do not show this decrease in amplitude. Rather more surprisingly, we found an alteration in the ability of *Vipr2^−/−^* SCN tissue to maintain a consistent circadian phase when cultured *in vitro*. WT SCN cultures expressed peak levels of PER2::LUC expression at a predictable projected CT phase, consistent with previous *in vivo* and *in vitro* investigations of *per2*/PER2 expression [47], [67]–[71]. Further, the earlier peak phase we observed for WT cultures prepared during the circadian day is consistent with a small phase advance of rhythms in cultures prepared at this time [58], [72].

Conversely, the rhythms of SCN cultures from *Vipr2^−/−^* mice were reset by cull/culture procedure and consistently peaked at the same time after culture preparation, regardless of the circadian phase at which this procedure was performed, illustrating the critical role of VIP-VPAC_2_ signaling in the maintenance of robust SCN function. These data suggest that while the strong rhythm generating properties of the WT SCN, at the unicellular and network levels, are sufficient to maintain *in vivo* phase, the diminished circadian capabilities of the relatively weak and disorganized *Vipr2^−/−^* SCN are unable to resist the phase-shifting influences associated with culture preparation. This presents an interesting parallel with a recent description of *in vitro* PER2::LUC phase in embryonic mouse liver [73]. Here, the authors find the phase of embryonic liver cultures to be determined by cull/culture time, while the phase of PER2::LUC rhythms in maternal liver is determined by external Zeitgeber time.

### WT and *Vipr2^−/−^* MBH and Pituitary Oscillators are Differentially Sensitive to Phase Resetting by Culture Procedure

The tendency for stronger oscillators to maintain phase and weaker oscillators to reset to cull time is also seen across the Arc/ME/PT, DMH and pituitary oscillators. In WT tissue, the stronger of the MBH areas investigated here, the Arc/ME/PT, maintains an *in vitro* phase similar to a prior report of the expression of *per2* in these areas [74], whereas the weaker oscillator contained in the DMH is reset by cull/culture. Similarly, WT pituitary, which contains a strong oscillator of comparable strength and robustness to the WT SCN (based on rhythm amplitude and a lack of damping), maintains a consistent *in vitro* CT phase of peak PER2::LUC expression. *Vipr2^−/−^* pituitary, however, appears to neither maintain a steady phase predicted by locomotor rhythms, nor fully reset according to cull/culture time. This lack of resetting of the *Vipr2^−/−^* pituitary differs from the properties of the *Vipr2^−/−^* SCN and indicates a reduced importance of VPAC_2_ signaling for the maintenance of pituitary rhythms – supported by the absence of any significant alteration to period, amplitude or rate of damping in *Vipr2^−/−^* pituitary.

While the phase of *Vipr2^−/−^* DMH responds to culturing in a similar fashion to WT DMH tissue, it is intriguing that WT and *Vipr2^−/−^* Arc/ME/PT and pituitary are differentially sensitive to the phase-resetting stimuli associated to culture preparation. This inability of *Vipr2^−/−^* Arc/ME/PT and pituitary to maintain an *in vitro* phase that correlates with the phase of behavioral rhythms is surprising given that these tissues show no alteration in period, amplitude or rate of damping of PER2::LUC expression. This raises the possibility that VPAC_2_ signaling within these oscillators impacts on other aspects of rhythm strength/robustness that we have not detected here. Further, bioluminescence imaging studies and microdissections of the individual rhythmic components of the Arc/ME/PT complex (Arc, ME, PT and the ependymal cell layer of the 3^rd^ ventricle; [47]) will be necessary to elucidate the relative contribution of these oscillators to both rhythmicity of the Arc/ME/PT complex in *Vipr2^−/−^* mice and its increased sensitivity to phase-resetting compared to WT tissue. The increased sensitivity of *Vipr2^−/−^* Arc/ME/PT and pituitary oscillators to stimuli associated with culture preparation presents an intriguing parallel with the increased sensitivity of mice lacking VIP-VPAC_2_ signaling to photic stimuli [17], [75].

### Variable Responses of WT and *Vipr2^−/−^* Tissue to GRP Treatment

Responses to GRP treatment were variable, with no consistent genotype differences across the tissues examined, or tissue differences between genotypes. Surprisingly, neither WT nor *Vipr2^−/−^* SCN cultures responded to GRP treatment with an increase in the amplitude of PER2 expression, as has been shown previously [27], [29]. These studies, however, differ in crucial technical details to the current investigation, measuring electrical activity with chronic GRP infusion and measuring *per1* expression in neonatal tissue, respectively. Indeed, the failure of GRP to boost rhythm amplitude of clock gene expression *in vitro* has previously been reported in cultured extra-SCN brain tissue [76]. Our observation of increased WT DMH rhythm amplitude following GRP treatment is of note given the lack of previous reports of GRP binding sites/GRP receptor expression in the DMH. This response is presumably mediated by previously undetected bombesin-like peptide receptors [77] in this area.

### Summary

The data presented here demonstrate that SCN and extra-SCN circadian oscillations are maintained in the absence of VIP-VPAC_2_ signaling. However, the *Vipr2^−/−^* tissues assessed here are differentially capable, compared to WT tissues, of maintaining a consistent circadian phase when cultured *in vitro*. Most surprisingly, the phase of PER2 oscillations in *Vipr2^−/−^* SCN is reset by culture preparation. Despite the maintenance of period, amplitude and rate of damping in the Arc/ME/PT, DMH and pituitary, the differential resetting effects of culture preparation on WT and *Vipr2^−/−^* phase in these areas presumably reflects circadian deficiencies not assessed here which may contribute towards the aberrant behavior and metabolism associated with *Vipr2^−/−^* animals.
